# Molecular Biology of Spermatozoa

**DOI:** 10.3390/ijms21093060

**Published:** 2020-04-26

**Authors:** Fernando J. Peña

**Affiliations:** Laboratory of Equine Reproduction and Equine Spermatology, Veterinary Teaching Hospital, University of Extremadura, 10003 Cáceres, Spain; fjuanpvega@unex.es; Tel.: +34-927-257167; Fax: +34-927-257102

The spermatozoon is a very special cell; it is generated in the male reproductive tract and has to travel to the female reproductive tract, of another individual, to fertilize an egg. However, through this amazing journey, it has to be able to respond and adapt to dramatically changing environments. The last decade has witnessed huge advances in the understanding of the molecular biology of this particular cell; these findings have provided new clues to understanding male infertility and the impact of reproductive technologies on sperm function. Thus, empirical approaches for sperm conservation are being substituted by approaches based in translational research. As a transcriptionally silent cell, spermatozoa largely depend on post-translational modifications of proteins to regulate their functions. Among these regulatory mechanisms, redox regulation plays a significant role, and the deregulation of redox homeostasis is behind male factor infertility. With the incorporation of “omics”, lipidomics, epigenomics, proteomics, and metabolomics, to the study of spermatozoa, knowledge regarding sperm function has grown significantly over the last decade. Developments in image analysis and advanced flow cytometry have also significantly contributed to this advance. The first part of the special issue of the International Journal of Molecular Sciences dedicated to the molecular biology of the spermatozoa has successfully published 11 outstanding papers, three reviews, and nine research articles that, in only one year, have received numerous citations, demonstrating the interest that has been raised in the scientific community. We are in the “omics” era, which provides very potent tools used increasingly in the areas of cell biology and medicine. In the study by Perez-Patiño et al. [1], changes in the proteome during the cryopreservation process of pig spermatozoa were studied. Cryopreservation, a technology widely used in animal breeding and human medicine, altered proteins directly involved in mitochondrial functionality. Spermatozoa are cells that show a high level of compartmentalization. The distribution of integrins that are transmembrane receptors involved in intracellular signaling and cell to cell adhesion was investigated in mouse spermatozoa [2], revealing that they may play specialized roles in sperm-epithelium and sperm-egg interaction. The interaction of the ejaculate with the female endometrium was studied in a porcine model [3]. This study showed that the ejaculate modified gene expression in the endometrium and oviduct, and this was dependent on the sperm fraction considered. The effect of leukemia in sperm biology was studied in a murine model [4], showing that leukemia altered spermatozoa, increasing the spontaneous acrosome reaction, and decreasing the number of pups born. Ethanol is one of the major concerns in public health and is directly linked to numerous forms of cancer and mental problems. In another study published in this *Special Issue,* the impact of ethanol on Sertoli cells was studied [5]. Capacitation is the process of maturation which spermatozoa undergo in the female genital tract, and new aspects of this mechanism were studied in a murine model showing new roles for 17 β-estradiol in this important mechanism for sperm maturation [6]. Aggressive chemotherapy leads to male infertility, so the development of methods to achieve in vitro spermatogenesis will be of great interest and has been the subject of one of the papers of this *Special Issue* [7]. Finally, a potential molecular marker of sperm quality is reported in the last research paper published [8], also stressing the importance of post-transcriptional modification of sperm proteins. In addition, three noteworthy reviews were published [9,10,11], covering interesting topics, including the role of taste receptors and the role of Zinc in sperm biology. A third review covered the mechanisms of sperm damage and repair, stressing the main role of spermatozoa as a transporter of DNA.
